# Characterization of the F-Box Gene Family and Its Expression under Osmotic Stress in Birch

**DOI:** 10.3390/plants12234018

**Published:** 2023-11-29

**Authors:** Guanbin Lv, Jingjing Shi, Jingnan Li, Guifeng Liu, Chuanping Yang, Jing Jiang

**Affiliations:** State Key Laboratory of Tree Genetics and Breeding, Northeast Forestry University, Harbin 150036, China; 18435155993@163.com (G.L.); 15714604842@163.com (J.S.); 18947655244@163.com (J.L.); liuguifeng@126.com (G.L.); yangcp@nefu.edu.cn (C.Y.)

**Keywords:** *Betula platyphylla* Su, BpF-box, osmotic stress, hormone

## Abstract

The F-box gene family is abundant in plants and crucial for plant growth and development. However, two questions prevail: Which F-box genes are involved in regulating plant biological processes? How do these genes regulate such biological processes? In this study, we characterized the F-box family and identified 240 *F-box* genes in birch (*Betula platyphylla* Suk.) via HMMER analysis. According to the C-terminal conserved domains, the F-box members were divided into 10 subfamilies. Through phylogenetic analysis, the F-box proteins were clustered into eight evolutionary branches. Synteny analyses suggested that the birch F-box gene family exhibits tandem and segmental duplication events. GO annotation analysis revealed that BpF-box proteins respond to stimuli, and regulate the defense response. According to RNA-Seq analysis, we found that 11 differentially expressed genes (DEGs) are responsive to osmotic stress. We performed co-expression analysis on the representative genes, and GO enrichment analysis further revealed that representative plant genes participate in the regulation of hormones, growth, and development. Through qRT-PCR, we found that the representative *BpF-box* genes are mainly involved in hormone response signaling pathways. It appears that the F-box gene family plays a significant role in the regulation of birch osmotic stress responses through the regulation of different hormones. Our results provided novel insights into the biological function of BpF-box proteins.

## 1. Introduction

Protein degradation is an important post transcriptional regulation process. Cells regulate key protein levels to swiftly respond to intracellular signals and changing environmental conditions [1]. In plants, the Ub/26S proteasome pathway is crucial for protein degradation [2]. The Ub/26S proteasome pathway relies on three enzymes—E1 (ubiquitin activation), E2 (ubiquitin binding), and E3 (ubiquitin ligase)—for the continuous process of ubiquitin activation, binding, and ligase activity, resulting in the attachment of ubiquitin subunits to specific target proteins [3]. The function of E3 in the ubiquitin–proteasome pathway is to recognize target proteins for ubiquitination [4], and the SCF protein complex composed of F-box, CULLIN, SKP1, and RBX1 proteins is the main type of E3 ubiquitin–protein ligases [3]. F-box proteins specifically recognize different substrates, and SCF complexes determine the target substrate for UPS degradation by binding to different F-box proteins [5].

The F-box gene family is abundant in plants [6,7], and F-box proteins have been identified in many plants; there are 694 genes in *Arabidopsis* [1], with 509 genes in soybean (*Glycine max*) [8], with 226 genes in pear (*Pyrus*) [9], and 193 genes in *S. alfredii* (*Sedum alfredii*) [10]. The N-terminus of the F-box protein has one or more F-box motifs, which are composed of approximately 50 amino acids and serve as sites for protein interactions [11]. TaFBA1 is an F-box protein, and Y2H experiments have shown that TaFBA1 can interact with SKP1, indicating that TaFBA1 is a subunit of the SCF complex [12]. The Arabidopsis F-box protein TLP can interact with specific Arabidopsis Skp3 (ASK) proteins [13]. Usually, the F-box domain at the N-terminus of the F-box protein binds to the Skp subunit, while its C-terminus domain can recognize different target proteins [1]. According to the different C-terminal domains, the F-box family can be divided into multiple subfamilies, which include 10 subfamilies in rice, with the largest subfamily being FBX [12]; and 9 subfamilies in soybeans [8].

The functions of the F-box protein are extensive, with involvement in participating in hormone regulation [14,15]. For example, PslSLY1 is an F-box protein that participates in the GA signal transduction of plums, restoring the plant GA signaling mechanism by overexpressing PslSLY1 in Arabidopsis sly1.10 [16]. The F-box protein AtSAGL1 (the Kelch repeat sequence) interacts with AtSARD1 and promotes SARD1 degradation through a 26s protease, negatively affecting SA synthesis in *Arabidopsis* [17]. Additionally, F-box proteins are involved in plant growth and development as well as physiological and biochemical reactions [18], including participating in plant stress responses to both biological and abiotic stresses [8,19,20]. In plants, the F-box gene mainly promotes the stress response by triggering different pathways and signaling networks. The F-box protein AtFBA1 increases tolerance to salt and osmotic stress by triggering ABA-mediated plant responses [21]. In *Arabidopsis*, the F-box protein AtFOF2 plays an important negative role in mediating seed germination and early seedling development by inhibiting the expression of ABA signaling genes ABI3 and ABI5; however, when AtFOF2 is overexpressed, higher ABA content is observed, which plays a positive role in plant drought tolerance [22]. The wheat F-box protein TaFBA-2A negatively regulates JA biosynthesis and enhances the salt tolerance of transgenic rice [21]; the F-box protein MAX2 contributes to the resistance of *Arabidopsis* to bacterial and plant pathogens [23]. In cotton, the negative regulation of plant osmotic stress occurs by the interaction between GhSKP1A and the F-box protein GhTULP34 [24]. Numerous F-box proteins have been discovered across different plant genomes, but the studies on the F-box proteins of birch are limited.

Birch is a deciduous broad-leaved tree species with fast growth, strong cold tolerance, and certain medicinal values. Therefore, it is crucial to comprehend the mechanisms of biotic and abiotic stress in birch. Many studies have found that the F-box protein can improve the adaptability of plants to the external environment [22,25]. However, a systematic analysis of the birch F-box gene family is lacking. In this study, we obtained 240 BpF-box genes via phylogenetic analysis, chromosome distribution, gene duplication, etc. We investigated the effect of osmotic stress on the expression of F-box genes. The key genes were analyzed through gene co-expression and network analysis, then we performed enrichment analysis on these genes. Finally, the expression patterns of these key genes under different hormone treatments were explored by us.

## 2. Results

### 2.1. Identification of the BpF-Box Family and Phylogenetic Tree

**Figure 1:**
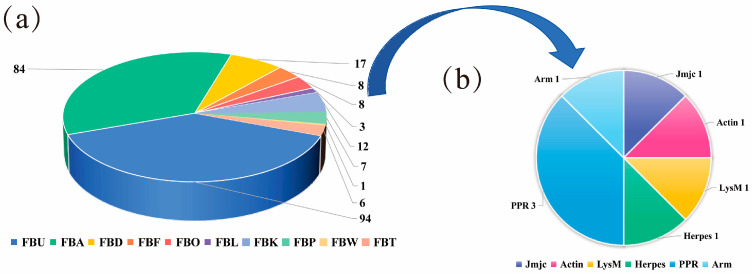
BpF-box protein classification based on C-terminal domains. (**a**) BpF-box proteins were categorized into ten subfamilies based on their C-terminal domains. (**b**) The FBO subfamily composition is further expanded and represented by the second pie chart, which shows the number of F-box proteins in each group. Different colors display different subfamilies.

Through HMMER analysis (E-value < 1 × 10^−3^), we identified 240 BpF-box genes in birch. The domain of the BpF-box proteins is shown in Figure 1 and Table S1. The results showed that 94 out of the 240 BpF-box genes contained only the F-box domain, 84 genes contained the FBA domain, 17 genes contained the FBD domain, 12 genes contained the FBK domain, 8 genes contained the FBF domain, 7 genes contained the FBP domain, 6 genes contained the FBT domain, 3 genes contained the FBL domain, 1 gene contained the FBW domain, and 16 FBO genes contained other structural domains, such as PPR (3), Jmjc (1), Actin (1), LysM (1), Herpes (1), and ARM (1).

**Figure 2:**
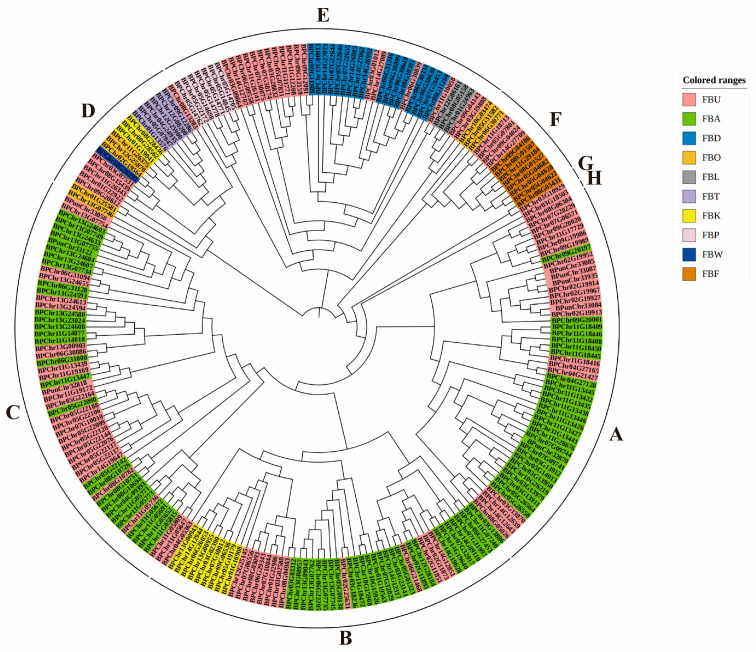
Dendrogram of BpF-box protein members was constructed using MEGA7 with the neighbor joining method. Distinct groups are indicated by different colors.

Figure 2 shows the constructed phylogenetic tree based on the BpF-box protein sequence. All BpF-box proteins were clustered into eight branches. FBU proteins were in all branches; FBO proteins were in four branches (A–H); FBA proteins were in three branches; FBK and FBF proteins were in two branches; and FBD, FBP, FBT, FBW and FBL proteins were in one branch. The results indicated a wider phylogenetic relationship for these proteins.

### 2.2. Gene Sequence Analysis and the Prediction of Cis-Elements

In this study, we examined the conservative motifs in 240 birch proteins via MEME software [26]. As shown in Figure S1a, 10 conserved protein motifs were obtained, and the prediction results showed that all members of groups A, B, and C have most motifs except motif 10. The members of groups D, E, and F have fewer motifs, and motif 10 only exists in group E. A group of closely related proteins had similar conservative motifs, which indicates that these proteins have similar functions. The motif sequence can be seen in Table S2.

The upstream 2000 bp sequences of the 240 *BpF-box* genes were extracted via TBtools. We used to Plant CARE (http://bioinformatics.psb.ugent.be/webtools/plantcare/html/, accessed on 10 July 2023) to analyze the *BpF-box* promoter and then obtained some cis-acting elements. As shown in Figure S1b, five hormone-related and five stress-related components were identified in 2 kb upstream regions of the *BpF-box* genes. Hormone-related elements include auxin-responsive and gibberellin-responsive elements, as well as salicylic acid, MeJA, and abscisic acid responsiveness. Stress-related elements include drought-inducibility and wound-responsive elements, as well as low temperature, defense, and stress responsiveness. Growth-related elements include meristem and endosperm expression, circadian control, and seed-specific regulation elements, as well as involvement in endosperm-specific negative expression, particularly in palisade mesophyll cells. According to these results, we hypothesized that the BpF-box family regulates hormone levels to impact plant defense against biotic and abiotic stresses.

### 2.3. Chromosome Distribution and Gene Duplication

**Figure 3:**
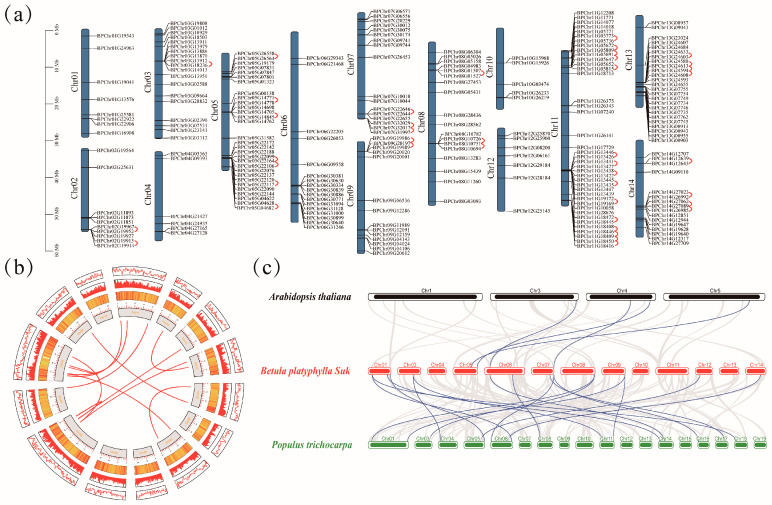
Chromosome distribution and gene duplication of BpF-box genes. (**a**) The BpF-box protein’s chromosome distribution spans Chr01–14, which corresponds to chromosome numbers 01–14. Red lines indicate gene pairs resulting from tandem duplication. (**b**) The BpF-box protein’s Collinearity analysis. Rectangles represent chromosomes 01–14. Gene density on the chromosomes was depicted through lines, heatmaps, and histograms. The analysis also revealed synteny blocks, depicted as gray lines in the birch genome, as well as segmental duplicated gene pairs represented by lines of various colors between chromosomes. (**c**) The BpF-box protein’s synteny analysis with *Arabidopsis* and poplar. Gray lines show gene blocks in birch that are orthologous to the other genomes, while blue lines mark the syntenic BpF-box pairs.

To identify the distribution of BpF-box genes on chromosomes, a birch database from CoGe was used. As shown in Figure 3a, 232 *BpF-box* genes were unevenly distributed on 14 chromosomes, and 8 *BpF-box* genes were not located on chromosomes. Chromosome 11 has the largest number of genes (40), followed by chromosomes 5 and 13, which have 31 and 26 genes, respectively. Chromosomes 1 and 10 have smaller distribution and contain 8 and 7 genes, respectively.

Gene duplication is crucial for the development of gene families. As shown in Figure 3a and Table S3, a total of 70 genes from chromosomes 2, 3, 5, 7, 8, 9, 11, 13, and 14 were classified as 41 tandem repeat events, with chromosome 11 having the most events (15). In addition, we also identified segmental duplication events, as shown in Figure 3b and Table S3. A total of 30 genes from chromosomes 1, 3, 6, 7, 8, 9, 11, 12, 13, and 14 were classified as 15 tandem repeat events, with chromosome 8 having the most events. These results suggest that some BpF-box genes could have originated from gene duplication, with segmental duplications potentially playing a significant role in BpF-box.

Regarding the *BpF-box* genes, we created a syntenic map by comparing sequence similarity between *Arabidopsis* and poplar (*Populus trichocarpa*). A total of 17 *BpF-box* genes are colinear with *Arabidopsis* and *Populus* L. Figure 3c and Table S4. Four *BpF-box* genes are collinear with four *Arabidopsis* genes. A total of 15 *BpF-box* genes are collinear with 23 *Populus* L. genes, which is far more than that between *Betula platyphylla* and *Arabidopsis*. This is probably because both *Betula platyphylla* and *Populus* L. are woody plants. We believe that in different species, these genes may have some important functions.

### 2.4. GO Annotation of BpF-Box Family Proteins

**Figure 4:**
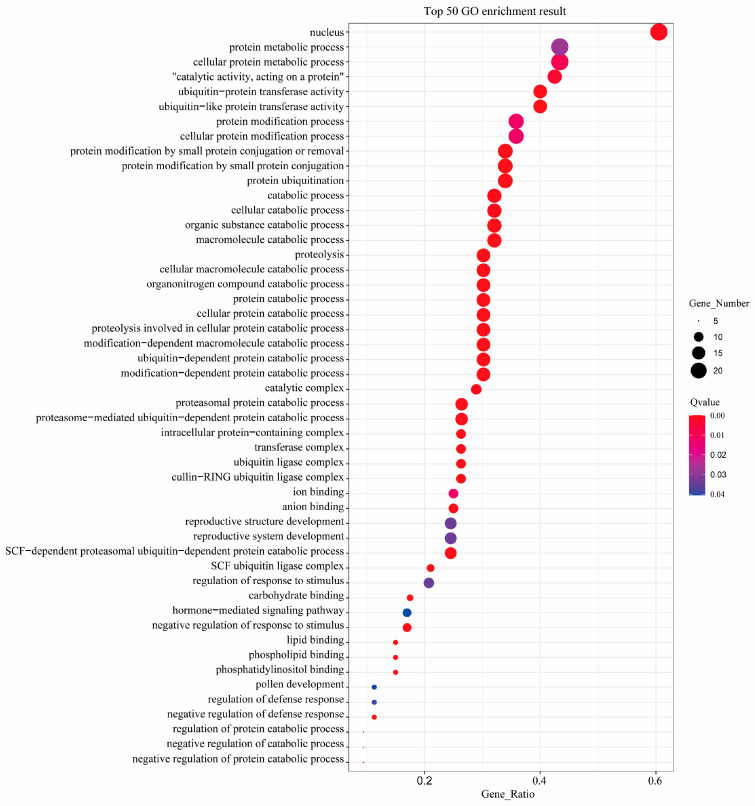
Go enrichment analysis of BpF-box members.

In order to better understand the involved pathway of the F-box family in birch, 243 F-box protein genes were predicted via the EggNOG database (http://eggnog-mapper.embl.de/, accessed on 15 August 2023). The results showed that F-box genes played crucial roles in molecular function, cellular components, and biological processes (Figure 4). F-box genes could be involved in many essential biological processes, including SCF-dependent proteasomal ubiquitin-dependent protein catabolic processes (GO:0031146), protein ubiquitination (GO:0016567), the negative regulation of the defense response (GO:0031348), the negative regulation of responses to stimuli (GO:0031348), the regulation of responses to stimuli (GO:0048583), the regulation of the defense response (GO:0031347), and hormone-mediated signaling pathways (GO:0009755).

### 2.5. The Expression of BpF-Box Family after PEG6000 Treatments

**Figure 5:**
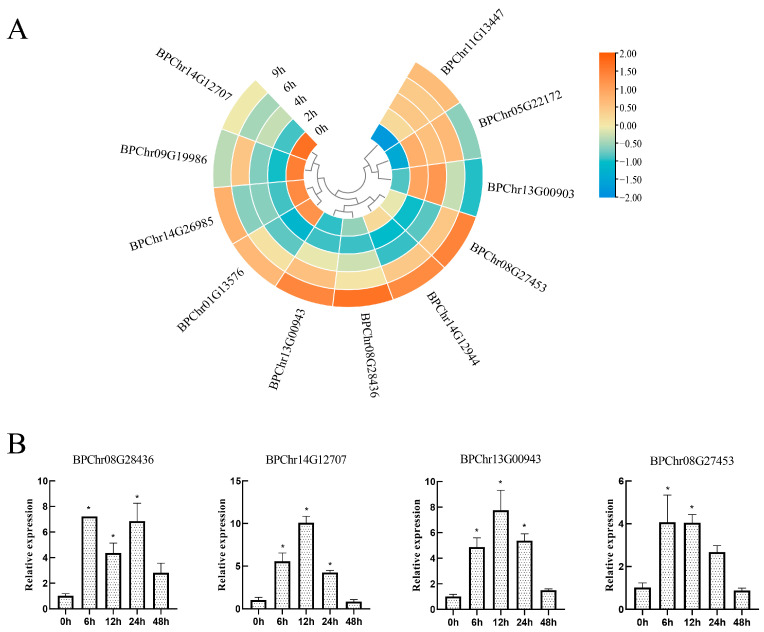
Differentially expressed F-box genes in birch. (**a**) Gene expression levels in response to PEG6000 stress based on RNA-Seq. (**b**) Gene expression levels in response to PEG6000 stress based on qRT-PCR. Error bars are standard deviations from the biologic replicates, asterisks indicate significant expression differences among different lines based on Student’s *t*-test (n = 3, *p* < 0.05).

We explored the expression of BpF-box genes after PEG6000 treatments by RNA-Seq. It was found that there were 11 DEGs after PEG6000 stress compared with the control (Figure 5a). Additionally, we found that three *BpF-box* genes were up-regulated, four *BpF-box* genes were down-regulated, and three *BpF-box* genes were negatively regulated at 2 h and as the duration of stress increased, the expression level of these three genes increased; there is another gene that was positively regulated at 2 h, but its expression decreased with the duration of stress.

We found that the expression levels of four genes—BPChr08G28436, BPChr13G00943, BPChr14G12707, and BPChr08G27453, increased with the duration of stress; therefore, these four genes were analyzed. The expression levels of four BpF-box genes in 6-week-old plants were measured under PEG6000 treatments for 0 h, 6 h, 12 h, 24 h and 48 h. The results showed that these genes were all positively correlated (Figure 5b).

### 2.6. Co-Expression Networks and Gene Ontology Analyses

**Figure 6:**
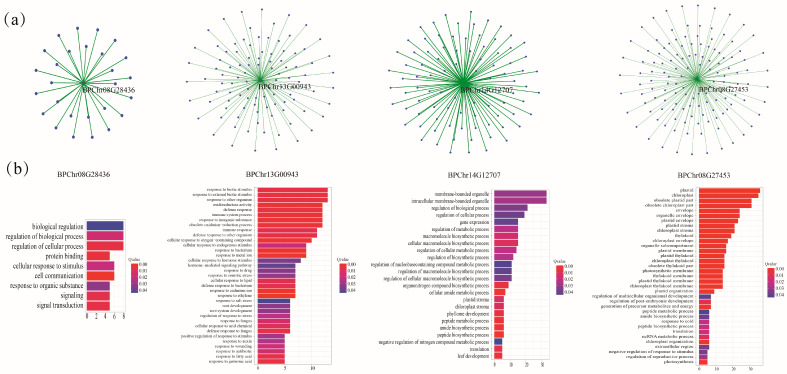
(**a**) Visual representation of co-expression networks for four differentially expressed genes, with dots representing genes and lines indicating their co-expression relationships. (**b**) Enrichment analysis of the co-expressed gene sets.

Co-expression analysis can help us find gene expression patterns with similar characteristics. These genes may affect the same physiological process or hormone pathway, and they are functionally related. After subjecting the transcriptome data to MeJA stress, we constructed a co-expression network consisting of four BpF-box genes. Consequently, we obtained four distinct co-expression networks (Figure 6a).

Four co-expressed gene sets were analyzed using gene set enrichment analysis (Figure 6b). These four *BpF-box* genes contain some GO terms, such as response to signal transduction, hormone, stimulus, stress, and metabolic process. This indicated that four *BpF-box* genes play an important role in the stress response. Therefore, we speculate that during the growth process of birch, BpF-box genes play a vital role in response to external stress.

### 2.7. BpF-Box Genes Expression under Different Hormone Treatments

**Figure 7:**
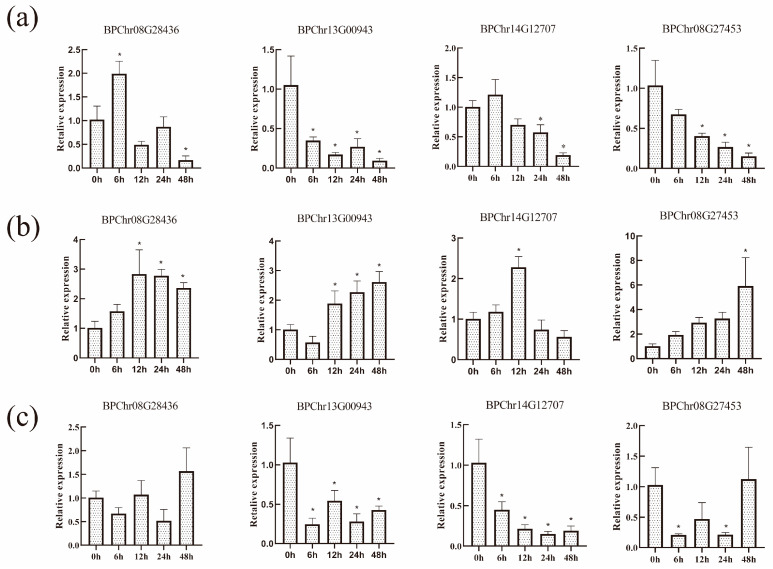
Gene expression levels of BpF-box under different hormone treatment. (**a**) Gene expression levels after MeJA treatment. Error bars are standard deviations from the biologic replicates. (**b**) Gene expression levels after ABA treatment. Error bars are standard deviations from the biologic replicates. (**c**) Gene expression levels after SA treatment. Error bars are standard deviations from the biologic replicates. Asterisks indicate significant expression differences among different lines based on Student’s *t*-test (n = 3, *p* < 0.05).

To better explore the functions and expression levels of the four *BpF-box* genes, 6-week-old plants were treated with MeJA, SA, and ABA for 0 h, 6 h, 12 h, 24 h and 48 h. The expression levels of the four *BpFbox* genes showed that there were different trends at different time points under MeJA stress. Three *BpF-box* genes (BPChr13G00943, BPChr14G12707, and BPChr08G27453) were all negatively correlated, but one *BpF-box* gene (BPChr08G28436) was positively correlated between 0 and 6 h and negatively correlated between 6 h and 48 h (Figure 7a). The expression levels of the four *BpF-box* genes under ABA stress showed different trends at different time points, and they were all regulated by ABA stress. The four *BpF-box* genes were all positively correlated (Figure 7b). Under SA treatment, the three *BpF-box* genes (BPChr13G00943, BPChr14G12707, and BPChr08G27453) were negatively correlated and one *BpF-box* gene (BPChr08G28436) did not respond to SA stress (Figure 7c). Combined with the results of the MeJA, SA, and ABA treatments, we believe that these *BpF-box* genes are involved in the regulation of the MeJA, ABA, and SA pathways.

## 3. Discussion

The F-box protein gene family is one of the largest gene families in plants. And previous research has shown that F-box proteins have been reported in plants such as wheat (*Triticum aestivum*) [7], soybean (*Glycine max*) [8], cotton (*Gossypium hirsutum L.*) [27], and *S. alfredii* (*Sedum alfredii* Hance) [10]. In this study, we identified 240 F-box genes in birch. In general, the F-box domain can bind to SKP1 to form an SCF complex, while the C-terminus domain of the F-box protein is responsible for identifying the target protein to be degraded, which is due to the different C-terminus domains. F-box proteins play different roles in plant growth and development [6]. By studying the C-terminus region of BpF-box, we were able to divide the F-box proteins into 10 subfamilies, with the FBU subfamily containing the most C-terminus genes and the other subfamilies being FBA, FBD, FBF, FBK, FBL, FBP, FBT, FBW, and FBO, which is consistent with previous research results [8,27]. We further classified the FBO subfamily and found that it contains six small subfamilies. Next, we conducted evolutionary tree analysis on the BpF-box proteins and found that subgroups with the same domain were roughly grouped together; however there were some exceptions, especially, the clustering of *BpF-box* subfamily genes, which is relatively chaotic, with some clustered together on their own and others clustered together with other subfamilies, indicating that the evolutionary history of BpF-box proteins may be complex [6].

The chromosome distribution results showed that 232 *BpF-box* genes are unevenly distributed on 14 chromosomes. In the process of plant evolution, gene families typically undergo tandem or large-scale segmented replication to produce a large number of genes [28,29]. Previous studies have found that in *Arabidopsis*, rice, and maize, some *F-box* genes are generated through tandem replication and segmental repetitive events [18,30]. Thus, tandem duplication and segmental duplications have played a role in the expansion of the F-box superfamily [6]. We investigated the tandem and segmental repeat events of the *BpF-box* genes, and the results showed that there were 41 tandem repeat events, with most of them occurring in its subfamily, but there were also a small number of tandem repeat events between FBU and FBA, as well as between FBU and FBD, this evidence indicating that the amplification of genes caused by tandem repeats mostly affects genes within in its subfamily. There are 15 gene pairs with fragment duplication events, which involve many subpopulations of genes without obvious patterns; these results indicated that some *BpF-box* genes originate from fragment duplication. We investigated the collinearity relationship between the BpF-box gene of birch and some genes in *Arabidopsis* and poplar. Four birch F-box genes have collinear relationships with other species, including one in *Arabidopsis* and six in poplar. The logarithm of collinearity genes between birch and poplar trees shows a much greater similarity compared to *Arabidopsis*, suggesting a strong genetic relationship of the F-BOX gene between the two tree species. Our research findings provide insights into the evolutionary relationship of the BpF-box family genes in birch.

The F-box protein participates in multiple pathways in plants [8]. It can affect the development of plant organs, seed germination, leaf senescence, and some biological, abiotic stresses, as well as participate in some signal transduction in plants [18]. However, the function of the *BpF-box* gene is unknown, so we conducted GO annotation on the *F-box* gene in birch for functional prediction, and the results showed that *BpF-box* genes played crucial roles in biological processes, such as regulation of response to stimuli and the defense response. The C-terminal of the F-box protein is used for specifically binding substrates to form SCF protein complexes, which affect the regulation of hormones or plant morphogenesis in plants, thereby regulating their tolerance to stress [2]. The interaction between the pepper F-box protein CaDIF1 and SKP 1 protein CaDIS1 enhances plant drought resistance by regulating ABA signaling [31]. In the GO annotation of the *BpF-box* genes, we found multiple occurrences of SCF-dependent prototypical annotations and ubiquitin ligase complex annotations. Therefore, we believe that the *BpF-box* genes play a role in the formation of SCF complexes and the ubiquitination process, which is consistent with some known research results [11,32].

An increasing amount of evidence suggests that F-box proteins and their SCF complexes also play a crucial role in regulating plant biological and abiotic stress responses, in addition to plant growth and development [2]. We analyzed the RNA Seq data from previous studies in order to study the *BpF-box* genes that respond to PEG6000 stress [33]. We found that five proteins responded to PEG6000 stress at three time points. Among these five genes, we found that the expression levels of two genes decreased over time, while the expression levels of three genes were upregulated over time. Therefore, we believe that these three genes play a more important role in birch osmotic stress. We further analyzed their expression levels and found that all three genes were positively correlated under osmotic stress.

We analyzed the co- expression network of three proteins, and then enriched the co-expression genes of these proteins. Through the above analysis, it was found that these F-box genes in *Betula platyphylla* are not only related to response to stress, but also to plant growth and development and plant hormones. We found that these genes are related to plant hormones, growth, and development. The protein degradation of the ubiquitin proteasome system (UPS) is an important mechanism for achieving biological development and function [2]. UPS requires ubiquitin molecules to adhere to target proteins for protein degradation [6], The F-box protein binds to the substrate protein through the protein interaction domain at the C-terminus and degrades the protein through UPS [34]. The different C-terminus domains result in F-box proteins recognizing different substrates, thereby enabling them to perform different functions. Through gene set enrichment analysis, we also found that the four key genes have their own specific functions, BPChr08G28436 (FBK) is mainly involved in cell communication and signal transduction, BPChr13G00943 (FBK) is mainly involved in response to hormone and response to stress, BPChr14G12707 (FBU) is mainly involved in regulation of biosynthetic processes, and BPChr08G27453 (FBA) is mainly focused on Cellular components.

The F-box protein is the main component of E3 ligase and is a receptor for various hormones, such as JA and ABA [3,35,36]. The response of plants to drought stress is coordinated by hormone signaling pathways [37,38,39]. These three genes were treated by ABA, SA, and MeJA; we validated the expression levels of these genes, and found that these F-box genes respond to hormone treatments. Plant hormones are crucial for regulating the interactions between plants and their complex biological and abiotic environments. Although various hormones have specific pathways, different hormone pathways may interfere with each other and have antagonistic, additive, and synergistic effects [40]. The *Arabidopsis* F-Box protein AtFBS1 has a certain degree of influence on JA- and ABA-related genes, indicating that it regulates the balance between these two important stress hormones to a certain extent [41]. Our research found that BPChr08G28436 was mainly related to the ABA pathway, BPChr14G12707 was related to the SA pathway, and BPChr13G00943 and BPChr08G27453 were related to ABA, SA, and MeJA. BPChr08G28436 and BPChr14G12707 respond to stress by regulating a single hormone. BPChr13G00943 and BPChr08G27453 respond to stress by regulating multiple hormones. We believe that the BpF-box genes are mainly involved in stress response by forming complexes with related proteins, thereby participating in protein degradation and affecting hormone signaling pathways, different F-box proteins play important roles in specific biological functions.

## 4. Materials and Methods

### 4.1. The Member of BpF-Box Genes and Construction of Phylogenetic Tree

The sequences of birch proteins were downloaded from (https://pubmed.ncbi.nlm.nih.gov/33574224/ 20 August 2023) [42]. The hidden Markov model (HMM) profiles of F-box (PF00646), F-box-like (PF12937), F-box-like 2 (PF13013), FBA (PF04300), FBA_1 (PF07734), FBA_2 (PF07735), FBA_3 (PF08268), and FBD (PF08387) [43] were obtained from the Pfam database (http://pfam.xfam.org/ 20 August 2023). To further screen the conserved domains of the F-box family, we used the SMART databases for further verification (SMART: Sequence analysis results) to test (E value < 1 × 10^−3^). The F-box proteins without F-box domain and redundant protein genes were removed. The SMART databases (SMART: Sequence analysis results) for verification to test were used to identify the domain in which located at the N-terminal is located (E-value < 1 × 10^−3^).

A Phylogenetic tree was constructed based on the amino acid sequences of *Betula platyphylla* suk via MEGAX using the neighbor joining method with 1000 repeated bootstrap tests [44]; the parameters are as follows: 1000 iterations of the bootstrap values and JTT (Jones–Taylor–Thornton) + G (Gamma Distributed) model, Gaps/Missing Data Treatment Optional partial deletion of 50% of the threshold.

### 4.2. Classification and Sequence Analysis on the BpF-Box Members

The genome sequences to analyze the BpF-box proteins were retrieved from (https://pubmed.ncbi.nlm.nih.gov/33574224/, accessed on 10 July 2023) [42]. The motifs in the BpF-box proteins were identified by the MEME Suite [26].

The birch promoter sequences were retrieved from (https://pubmed.ncbi.nlm.nih.gov/33574224/, accessed on 10 July 2023) [42]. The Plant CARE online website (http://bioinformatics.psb.ugent.be/webtools/plantcare/html/, accessed on 21 July 2023), was used it to analyze the birch *BpF-box* promoters [45]. TBtools (V2.008 software)was used to visualize the promoter elements [46].

### 4.3. Chromosome Distribution and Gene Duplication

The birch genome data were downloaded from the Phytozome database (https://phytozome.jgi.doe.gov/, accessed on 5 August 2023). According to the information from the GFF3 file in the Phytozome database, we obtained the detailed chromosome location of each *BpF-box* gene. *BpF-box* genes location on chromosomes was visualized using the annotation information of the birch genome via TBtools [46]. We analyzed the tandem duplication events of the *BpF-box* genes and investigated segmental duplication events by using MCScanX and BLASTP methods in TBtools [47].

Protein sequences and GFF3 annotation files of *A. thaliana*, *Populus trichocarpa* were downloaded from Phytozome database. The Dual Synteny Plotter package was used to calculate and visualize the collinear relationship of homologous proteins of *Betula platyphylla* and other species.

### 4.4. BpF-Box Proteins and GO Functional Annotation in Birch

The EggNOG database (http://eggnog-mapper.embl.de/, accessed on 15 August 2023) was used for the functional prediction of F-box genes [48], and TBtools was used for gene ontology enrichment [46]. The *p*-value of each pathway was calculated and adjusted according to the method of the Benjamin–Hochberg method [49].

### 4.5. Gene Expression Analysis

To characterize the response of the *BpF-box* genes to PEG6000 in birch, we analyzed the RNA-Seq data from previous studies [50]. These data contain the gene expression levels of birch treated with 150 mM PEG6000 for 2 h, 4 h, 6 h and 9 h. Plants irrigated with water were used as the control. The differentially expressed genes (DEGs) were identified using DESeq software and the thresholds were fold change ≥ 2 and *p*-value adjusted (padj) for multiple tests < 0.05 [51].

### 4.6. Using qRT-PCR to Validate Differentially Expressed Genes

Wild-type birch seedlings were cultivated and grown under laboratory conditions for one month. Then, birch seedlings were divided into five equal groups, but ensuring that each group grew similarly. Three plants were used for biological replication. Every group was treated with 150 mM PEG6000 for 6 h, 12 h, 24 h and 48 h and the control seedlings were irrigated with water. We used qRT-PCR to verify the gene expression levels before and after PEG6000 treatment and treated them with 150 mM PEG6000 for 0, 6, 12, 24 and 48 h. qRT-PCR experiment was performed using 7500 real-time fluorescent quantitative PCR instrument (ABI) with SYBR Green real-time PCR Master Mix (TOYOBO, OSAKA, Japan). The reaction system and reaction procedure were carried out according to the product protocol (TOYOBO, OSAKA, Japan). The 2^−ΔΔCt^ method was used to analyze the gene expression of BpF-box [52].

### 4.7. Gene Co-Expression Networks and Gene Ontology Analyses

We constructed a co-expression network for key differentially expressed genes using the Spearman method [53]. We selected genes with a correlation coefficient greater than 0.9 with the BpF-box gene to construct a co-expression network, and the Pearson algorithm was used to calculate the correlation coefficient between genes, The co-expression network was visualized using Cytoscape [54]. The TBtools was used to study the gene set enrichment analysis (*p*-value < 0.05).

### 4.8. BpF-Box Genes Expression under Different Hormone Treatments

Wild-type birch seedlings were cultivated and grown under laboratory conditions for one month. Then, the birch seedlings were divided into three groups, ensuring that each group grew similarly. The control seedlings were irrigated with water and each of the group was then treated with 100 μmol/L MeJA, 100 μmol/L SA, and 100 μmol/L ABA for 6 h, 12 h, 24 h, and 48 h, respectively. Three plants were used for biological replication in both the control treatment and four time periods of the hormone treatment. Subsequently, RNA was extracted from the sample and then reverse transcribed into cDNA. Finally, the gene expression of BpF-box was obtained through qRT-PCR. Primer sequences for qRT-PCR were listed in Table S5. The 2^−ΔΔCt^ method was used to analyze the gene expression of BpF-box [52].

## 5. Conclusions

In this study, we identified 240 F-box gene family members in birch and characterized their conserved F-box domains. We then conducted systematic analyses of the *BpF-box* gene family. The *BpF-box* family members were divided into 10 subfamilies. Through phylogenetic analysis, the F-box proteins were clustered into eight evolutionary branches. The *BpF-box* promoter contained hormone-related, stress-related, and growth-related cis-acting elements. A total of 240 *BpF-box* genes were unevenly distributed on 14 chromosomes. A total of 70 *BpF-box* genes formed 41 tandem repeat events, and a total of 30 *BpF-box* genes from chromosomes 1, 3, 6, 7, 8, 9, 11, 12, 13 and 14 were classified as 15 tandem repeat events. Additionally, we explored the collinearity relationship between the BpF-box gene of birch and some genes in *Arabidopsis* and poplar. We identified PEG6000-inducible gene expression patterns. On the basis of the four key corresponding gene networks and qRT-PCR analysis, the key proteins were found to be involved in hormone response signaling pathways.

## Data Availability

Data available in a publicly accessible repository.

## Supplementary Materials

The following supporting information can be downloaded at: https://www.mdpi.com/article/10.3390/plants12234018/s1, Figure S1: Gene motif and promoter elements compositions of the BpF-box members; Table S1: Classification of F-box proteins in birch based on C-terminal domains; Table S2: Sequence of Motif Sequence; Table S3: Tandemly and segmentally duplicated BpF-box gene pairs; Table S4: Syntenic gene pairs; Table S5: Primer sequences for qRT-PCR.

## References

[1] Gagne J.M., Downes B.P., Shiu S.H., Durski A.M., Vierstra R.D. (2002). The F-box subunit of the SCF E3 complex is encoded by a diverse superfamily of genes in *Arabidopsis*. Proc. Natl. Acad. Sci. USA.

[2] Smalle J., Vierstra R.D. (2004). The ubiquitin 26S proteasome proteolytic pathway. Annu. Rev. Plant Biol..

[3] Vierstra R.D. (2009). The ubiquitin-26S proteasome system at the nexus of plant biology. Nat. Rev. Mol. Cell Biol..

[4] Patton E.E., Willems A.R., Tyers M. (1998). Combinatorial control in ubiquitin-dependent proteolysis: Don’t Skp the F-box hypothesis. Trends Genet. TIG.

[5] Jin J.P., Ang X.L.L., Shirogane T., Harper J.W., Deshaies R.J. (2005). Identification of substrates for F-Box proteins. Ubiquitin and Protein Degradation, Pt B.

[6] Xu G.X., Ma H., Nei M., Kong H.Z. (2009). Evolution of F-box genes in plants: Different modes of sequence divergence and their relationships with functional diversification. Proc. Natl. Acad. Sci. USA.

[7] Li H.Y., Wei C.R., Meng Y.Y., Fan R.Q., Zhao W.Q., Wang X.D., Yu X.M., Laroche A., Kang Z.S., Liu D.Q. (2020). Identification and expression analysis of some wheat F-box subfamilies during plant development and infection by *Puccinia triticina*. Plant Physiol. Biochem..

[8] Jia Q., Xiao Z.X., Wong F.L., Sun S., Liang K.J., Lam H.M. (2017). Genome-Wide Analyses of the Soybean F-Box Gene Family in Response to Salt Stress. Int. J. Mol. Sci..

[9] Wang G.M., Yin H., Qiao X., Tan X., Gu C., Wang B.H., Cheng R., Wang Y.Z., Zhang S.L. (2016). F-box genes: Genome-wide expansion, evolution and their contribution to pollen growth in pear (*Pyrus bretschneideri*). Plant Sci. Int. J. Exp. Plant Biol..

[10] Zhang Z., Qiu W.M., Liu W., Han X.J., Wu L.H., Yu M., Qiu X.L., He Z.Q., Li H.Y., Zhuo R.Y. (2021). Genome-wide characterization of the hyperaccumulator *Sedum alfredii* F-box family under cadmium stress. Sci. Rep..

[11] Kipreos E.T., Pagano M. (2000). The F-box protein family. Genome Biol..

[12] Li Q.X., Wang W.Q., Wang W.L., Zhang G.Q., Liu Y., Wang Y., Wang W. (2018). Wheat F-Box Protein Gene *TaFBA1* Is Involved in Plant Tolerance to Heat Stress. Front. Plant Sci..

[13] Bao Y., Song W.M., Jin Y.L., Jiang C.M., Yang Y., Li B., Huang W.J., Liu H., Zhang H.X. (2014). Characterization of *Arabidopsis* Tubby-like proteins and redundant function of AtTLP3 and AtTLP9 in plant response to ABA and osmotic stress. Plant Mol. Biol..

[14] Gao Y., Zhao Y., Li T.T., Liu Y., Ren C.X., Wang M.L. (2010). Molecular cloning and expression analysis of an F-box protein gene responsive to plant hormones in *Brassica napus*. Mol. Biol. Rep..

[15] Kepinski S., Leyser O. (2005). The *Arabidopsis* F-box protein TIR1 is an auxin receptor. Nature.

[16] El-Sharkawy I., Ismail A., Darwish A., El Kayal W., Subramanian J., Sherif S.M. (2021). Functional characterization of a gibberellin F-box protein, PslSLY1, during plum fruit development. J. Exp. Bot..

[17] Yu K., Yang W.Q., Zhao B., Wang L., Zhang P., Ouyang Y., Chang Y.K., Chen G.Z., Zhang J.L., Wang S.J. (2022). The Kelch-F-box protein SMALL AND GLOSSY LEAVES 1 (SAGL1) negatively influences salicylic acid biosynthesis in *Arabidopsis thaliana* by promoting the turn-over of transcription factor SYSTEMIC ACQUIRED RESISTANCE DEFICIENT 1 (SARD1). New Phytol..

[18] Yang X.H., Kalluri U.C., Jawdy S., Gunter L.E., Yin T.M., Tschaplinski T.J., Weston D.J., Ranjan P., Tuskan G.A. (2008). The F-Box Gene Family Is Expanded in Herbaceous Annual Plants Relative to Woody Perennial Plants. Plant Physiol..

[19] Stone S.L. (2014). The role of ubiquitin and the 26S proteasome in plant abiotic stress signaling. Front. Plant Sci..

[20] Paquis S., Mazeyrat-Gourbeyre F., Fernandez O., Crouzet J., Clément C., Baillieul F., Dorey S. (2011). Characterization of a F-box gene up-regulated by phytohormones and upon biotic and abiotic stresses in *grapevine*. Mol. Biol. Rep..

[21] Gao L.T., Jia S.Z., Cao L., Ma Y.J., Wang J.L., Lan D., Guo G.Y., Chai J.F., Bi C.L. (2022). An F-box protein from *wheat*, TaFBA-2A, negatively regulates JA biosynthesis and confers improved salt tolerance and increased JA responsiveness to transgenic rice plants. Plant Physiol. Biochem..

[22] Qu L., Sun M.S., Li X.M., He R.Q., Zhong M., Luo D., Liu X.M., Zhao X.Y. (2020). The *Arabidopsis* F-box protein FOF2 regulates ABA-mediated seed germination and drought tolerance. Plant Sci..

[23] Piisilä M., Keceli M.A., Brader G., Jakobson L., Joesaar I., Sipari N., Kollist H., Palva E.T., Kariola T. (2015). The F-box protein MAX2 contributes to resistance to bacterial phytopathogens in *Arabidopsis thaliana*. BMC Plant Biol..

[24] Li Z.S., Wang X.Y., Cao X.C., Chen B.Z., Ma C.K., Lv J.Y., Sun Z.M., Qiao K.K., Zhu L.F., Zhang C.J. (2021). GhTULP34, a member of tubby-like proteins, interacts with GhSKP1A to negatively regulate plant osmotic stress. Genomics.

[25] Kim Y.Y., Cui M.H., Noh M.S., Jung K.W., Shin J.S. (2017). The FBA motif-containing protein AFBA1 acts as a novel positive regulator of ABA response in *Arabidopsis*. Plant Cell Physiol..

[26] Bailey T.L., Boden M., Buske F.A., Frith M., Grant C.E., Clementi L., Ren J.Y., Li W.W., Noble W.S. (2009). MEME SUITE: Tools for motif discovery and searching. Nucleic Acids Res..

[27] Zhang S.L., Tian Z.L., Li H.P., Guo Y.T., Zhang Y.Q., Roberts J.A., Zhang X.B., Miao Y.C. (2019). Genome-wide analysis and characterization of F-box gene family in *Gossypium hirsutum* L.. BMC Genom..

[28] Cannon S.B., Mitra A., Baumgarten A., Young N.D., May G. (2004). The roles of segmental and tandem gene duplication in the evolution of large gene families in *Arabidopsis thaliana*. BMC Plant Biol..

[29] Zhao K., Chen S., Yao W.J., Cheng Z.H., Zhou B.R., Jiang T.B. (2021). Genome-wide analysis and expression profile of the bZIP gene family in *poplar*. BMC Plant Biol..

[30] Jia F.J., Wu B.J., Li H., Huang J.G., Zheng C.C. (2013). Genome-wide identification and characterisation of F-box family in *maize*. Mol. Genet. Genom..

[31] Lim J., Lim C.W., Lee S.C. (2019). Functional Analysis of Pepper F-box Protein CaDIF1 and Its Interacting Partner CaDIS1: Modulation of ABA Signaling and Drought Stress Response. Front. Plant Sci..

[32] Nguyen K.M., Busino L., Sun Y., Wei W., Jin J. (2020). The Biology of F-box Proteins: The SCF Family of E3 Ubiquitin Ligases. Cullin-Ring Ligases and Protein Neddylation: Biology and Therapeutics.

[33] Jiao J., Gao F., Liu J., Lv Z.Y., Liu C.M. (2022). A structural basis for the functional differences between the cytosolic and plastid phosphoglucose isomerase isozymes. PLoS ONE.

[34] Abd-Hamid N.A., Ahmad-Fauzi M.I., Zainal Z., Ismail I. (2020). Diverse and dynamic roles of F-box proteins in plant biology. Planta.

[35] Adams E., Turner J. (2010). COI1, a jasmonate receptor, is involved in ethylene-induced inhibition of *Arabidopsis* root growth in the light. J. Exp. Bot..

[36] Dharmasiri N., Dharmasiri S., Estelle M. (2005). The F-box protein TIR1 is an auxin receptor. Nature.

[37] Zhao Y., Chan Z.L., Gao J.H., Xing L., Cao M.J., Yu C.M., Hu Y.L., You J., Shi H.T., Zhu Y.F. (2016). ABA receptor PYL9 promotes drought resistance and leaf senescence. Proc. Natl. Acad. Sci. USA.

[38] Xiong B., Wang Y., Zhang Y., Ma M.M., Gao Y.F., Zhou Z.Y., Wang B.Z., Wang T., Lv X.L., Wang X. (2020). Alleviation of drought stress and the physiological mechanisms in *Citrus* cultivar (*Huangguogan*) treated with methyl jasmonate. Biosci. Biotechnol. Biochem..

[39] Dong Q.L., Duan D.Y., Zheng W.Q., Huang D., Wang Q., Yang J., Liu C.H., Li C., Gong X.Q., Li C.Y. (2022). Overexpression of *MdVQ37* reduces drought tolerance by altering leaf anatomy and SA homeostasis in transgenic apple. Tree Physiol..

[40] Aerts N., Mendes M.P., Van Wees S.C.M. (2021). Multiple levels of crosstalk in hormone networks regulating plant defense. Plant J..

[41] Gonzalez L.E., Keller K., Chan K.X., Gessel M.M., Thines B.C. (2017). Transcriptome analysis uncovers *Arabidopsis* FBOX STRESS INDUCED 1 as a regulator of jasmonic acid and abscisic acid stress gene expression. BMC Genom..

[42] Chen S., Wang Y.C., Yu L.L., Zheng T., Wang S., Yue Z., Jiang J., Kumari S., Zheng C.F., Tang B. (2021). Genome sequence and evolution of *Betula platyphylla*. Hortic. Res.-Engl..

[43] Gupta S., Garg V., Kant C., Bhatia S. (2015). Genome-wide survey and expression analysis of F-box genes in *chickpea*. BMC Genom..

[44] Kumar S., Stecher G., Li M., Knyaz C., Tamura K. (2018). MEGA X: Molecular Evolutionary Genetics Analysis across Computing Platforms. Mol. Biol. Evol..

[45] Lescot M., Déhais P., Thijs G., Marchal K., Moreau Y., Van de Peer Y., Rouzé P., Rombauts S. (2002). PlantCARE, a database of plant cis-acting regulatory elements and a portal to tools for in silico analysis of promoter sequences. Nucleic Acids Res.

[46] Chen C.J., Chen H., Zhang Y., Thomas H.R., Frank M.H., He Y.H., Xia R. (2020). TBtools: An Integrative Toolkit Developed for Interactive Analyses of Big Biological Data. Mol. Plant.

[47] Wang Y.P., Tang H.B., DeBarry J.D., Tan X., Li J.P., Wang X.Y., Lee T.H., Jin H.Z., Marler B., Guo H. (2012). MCScanX: A toolkit for detection and evolutionary analysis of gene synteny and collinearity. Nucleic Acids Res..

[48] Huerta-Cepas J., Szklarczyk D., Heller D., Hernández-Plaza A., Forslund S.K., Cook H., Mende D.R., Letunic I., Rattei T., Jensen L.J. (2019). eggNOG 5.0: A hierarchical, functionally and phylogenetically annotated orthology resource based on 5090 organisms and 2502 viruses. Nucleic Acids Res..

[49] Benjamini Y., Hochberg Y. (1995). Controlling the False Discovery Rate: A Practical and Powerful Approach to Multiple Testing. J. R. Stat. Soc. Ser. B.

[50] Jia Y.Q., Niu Y.I., Zhao H.M., Wang Z.B., Gao C.Q., Wang C., Chen S., Wang Y.C. (2022). Hierarchical transcription factor and regulatory network for drought response in *Betula platyphylla*. Hortic. Res.-Engl..

[51] Anders S., Huber W. (2010). Differential expression analysis for sequence count data. Genome Biol..

[52] Yao W.J., Wang S.J., Zhou B.R., Jiang T.B. (2016). Transgenic poplar overexpressing the endogenous transcription factor *ERF76* gene improves salinity tolerance. Tree Physiol..

[53] Pripp A.H. (2018). Pearson’s or Spearman’s correlation coefficients. Tidsskr. Nor. Laegeforen..

[54] Shannon P., Markiel A., Ozier O., Baliga N.S., Wang J.T., Ramage D., Amin N., Schwikowski B., Ideker T. (2003). Cytoscape: A software environment for integrated models of biomolecular interaction networks. Genome Res..

