# Supplementary material for: Characterization of the F-Box Gene Family and Its Expression under Osmotic Stress in Birch
Source: Plants (Basel). 2023 Nov 29;12(23):4018. doi: 10.3390/plants12234018 (PMC10707895; doi:10.3390/plants12234018)
Supplement: Supplementary file 1 [file plants-12-04018-s001.zip › Table S1.pdf]

Table S1 Classification of F-box proteins in birch based on C-terminal domains

FBU	FBA	FBD	FBO	FBL	FBT	FBK	FBP	FBW	FBF
FBU	FBA	FBD	FBO	LRR	TUB	kelch	PP2	WD40	DUF295
BPChr11G05647	BPChr03G23141	BPChr14G26992	BPChr01G25581	BPChr12G06161	BPChr12G08200	BPChr01G16908	BPChr05G14845	BPChr10G26233	BPChr12G29184
BPChr13G24594	BPChr14G19628	BPChr14G27023	BPChr06G30771	BPChr01G24963	BPChr12G25900	BPChr05G19179	BPChr05G14771		BPChr08G01527
BPChr02G25631	BPChr07G32029	BPChr07G22644	BPChr05G31582	BPChr08G05158	BPChr05G07847	BPChr14G12944	BPChr05G14698		BPChr05G04682
BPChr05G22076	BPChr03G13912	BPChr06G31246	BPChr02G19564		BPChr09G12286	BPChr09G06536	BPChr05G14778		BPChr08G01507
BPChr13G07754	BPChr11G05773	BPChr06G30630	BPChr10G03474		BPChr04G24935	BPChr13G00943	BPChr05G14705		BPChr05G04628
BPChr07G06571	BPChr11G05652	BPChr06G30381	BPChr13G07746		BPChr01G13576	BPChr07G30012	BPChr05G22172		BPChr08G05431
BPChr07G10010	BPChr04G09393	BPChr06G30899	BPChr12G25870			BPChr03G02390	BPChr05G14762		BPChr05G04622
BPChr05G22188	BPChr13G24684	BPChr06G30640	BPChr11G26375			BPChr08G28562			BPChr09G04106
BPChr05G22106	BPChr11G13435	BPChr06G30334				BPChr01G19041			
BPChr02G11873	BPChr11G13422	BPChr14G27062				BPChr08G28436			
BPChr08G06304	BPChr08G27453	BPChr14G26985				BPChr13G00955			
BPChr05G22099	BPChr11G13427	BPChr07G22646				BPChr06G26053			
BPChr08G04983	BPChr11G05815	BPChr07G26453							
BPChr06G21468	BPChr11G13445	BPChr07G22655							
BPChr13G24613	BPChr11G05691	BPChr09G11989							
BPChr11G19169	BPChr11G05672	BPChr08G11260							
BPChr14G12643	BPChr13G24593	BPChr13G00913							
BPChr11G05736	BPChr14G12317								
BPChr09G12091	BPChr03G14013								
BPChr11G13439	BPChr06G22205								
BPChr13G00903	BPChr13G07735								
BPChr08G05026	BPChr11G18446								
BPChr01G22906	BPChr11G13438								

BPChr02G19967	BPChr11G13426
BPunChr32818	BPChr12G28184
BPChr11G19172	BPChr11G05771
BPunChr33050	BPChr13G24607
BPunChr33935	BPChr11G18409
BPChr06G31094	BPChr13G07762
BPChr06G29343	BPChr11G13441
BPChr02G19914	BPChr11G07240
BPChr11G18416	BPChr07G09744
BPChr02G19927	BPChr07G31997
BPChr11G05809	BPChr13G07745
BPChr03G18503	BPChr05G01323
BPChr13G24655	BPChr13G23024
BPChr06G30839	BPChr09G20197
BPunChr33084	BPChr13G24603
BPChr02G19913	BPChr13G07734
BPChr05G22137	BPChr13G08957
BPunChr33087	BPChr10G15926
BPChr05G22164	BPChr07G09741
BPChr14G12639	BPChr03G13911
BPChr08G15439	BPChr11G18472
BPChr14G19647	BPChr08G10694
BPChr09G19986	BPChr13G09043
BPChr03G02588	BPChr10G15968
BPChr09G19989	BPChr02G11893
BPChr05G22144	BPChr08G10751

BPChr12G25145	BPChr04G27128
BPunChr33085	BPChr13G07749
BPChr06G30886	BPChr09G20001
BPChr03G01012	BPChr11G18445
BPChr05G22120	BPChr03G18236
BPChr08G10782	BPunChr32870
BPChr11G17729	BPChr05G00138
BPChr04G05363	BPChr03G13886
BPChr05G22117	BPunChr33122
BPChr11G18713	BPChr13G24608
BPChr03G10929	BPChr11G13447
BPChr07G10044	BPChr13G07755
BPChr08G03093	BPChr13G24633
BPChr03G09664	BPChr03G13979
BPChr14G12707	BPChr06G31000
BPChr03G19808	BPChr10G26219
BPChr02G11851	BPChr06G31128
BPChr03G13870	BPChr03G13950
BPChr07G30075	BPChr13G24580
BPChr11G26141	BPChr05G22090
BPChr14G27089	BPChr11G14077
BPChr01G22922	BPChr11G14018
BPChr11G10058	BPChr11G13446
BPChr09G20020	BPChr05G26564
BPChr09G12159	BPChr05G22142
BPChr14G12851	BPChr08G10726

BPChr02G19952	BPChr05G26558
BPChr07G06556	BPChr11G28676
BPChr07G20229	BPChr11G18450
BPChr11G26343	BPChr11G18408
BPChr09G04024	BPChr14G09110
BPChr06G09558	BPChr07G32017
BPChr05G07801	BPChr01G19543
BPChr14G27709	BPChr03G03312
BPChr04G27165	BPChr14G19640
BPChr03G07531	
BPChr07G30174	
BPChr04G21427	
BPChr08G13283	
BPChr09G04143	
BPChr05G07831	
BPChr09G20612	
BPChr11G12208	
BPChr03G28832	
BPChr11G11771	
BPChr13G21385	
BPChr05G22097	

Classification of FBO proteins in birch

FBO	FBL	FBT	FBK	FBP	FBW
JmJC	Actin	LysM	Herpes	PPR	ARM
BPChr01G25581	BPChr05G31582	BPChr10G03474	BPChr02G19564	BPChr13G07746	BPChr06G30771

BPChr12G25870

BPChr11G26375
