# Supplementary material for: Characterization of the F-Box Gene Family and Its Expression under Osmotic Stress in Birch
Source: Plants (Basel). 2023 Nov 29;12(23):4018. doi: 10.3390/plants12234018 (PMC10707895; doi:10.3390/plants12234018)
Supplement: Supplementary file 1 [file plants-12-04018-s001.zip › Table S2.pdf]

Table S2 Sequence of Motif Sequence

	Motif Sequence
Motif 1	LPEEVLVEILSRLPVKSLLRFRCVSKSWRS
Motif 2	FDIWVMKEYGVKESWTKLFTI
Motif 3	GFGFDPKTNDYKVVR
Motif 4	IVSFDMSDEVFREMPLPDSLV
Motif 5	PPLVEVYSLRTGSWR
Motif 6	IVLWNPAIRKSKTLP
Motif 7	LISSPBFISKHLNHSLSNNNY
Motif 8	LKIVGSCNGLLCLAB
Motif 9	PSVFLNGALHWLAYT
Motif 10	KRFNLCCDVLRDASRVNTWISAAVRHNVQ
