# Supplementary material for: Characterization of the F-Box Gene Family and Its Expression under Osmotic Stress in Birch
Source: Plants (Basel). 2023 Nov 29;12(23):4018. doi: 10.3390/plants12234018 (PMC10707895; doi:10.3390/plants12234018)
Supplement: Supplementary file 1 [file plants-12-04018-s001.zip › Table S3 .pdf]

Table S3Tandemly and segmentally duplicated BpF-box gene pairs.

Gene ID	Gene ID	Duplication Type
BPChr02G19967	BPChr02G19952	Tandem duplication
BPChr02G19913	BPChr02G19914	Tandem duplication
BPChr03G13912	BPChr03G18236	Tandem duplication
BPChr05G26558	BPChr05G26564	Tandem duplication
BPChr05G14771	BPChr05G14778	Tandem duplication
BPChr05G14705	BPChr05G14845	Tandem duplication
BPChr05G22099	BPChr05G22164	Tandem duplication
BPChr05G22164	BPChr05G22106	Tandem duplication
BPChr05G22120	BPChr05G22117	Tandem duplication
BPChr05G04628	BPChr05G04682	Tandem duplication
BPChr07G22646	BPChr07G22644	Tandem duplication
BPChr07G32029	BPChr07G32017	Tandem duplication
BPChr07G32017	BPChr07G31997	Tandem duplication
BPChr09G19986	BPChr09G20197	Tandem duplication
BPChr09G20197	BPChr09G19989	Tandem duplication
BPChr08G01507	BPChr08G01527	Tandem duplication
BPChr08G10726	BPChr08G10751	Tandem duplication
BPChr08G10751	BPChr08G10694	Tandem duplication
BPChr11G05773	BPChr11G05736	Tandem duplication
BPChr11G05672	BPChr11G05809	Tandem duplication
BPChr11G05809	BPChr11G05691	Tandem duplication
BPChr11G05691	BPChr11G05647	Tandem duplication
BPChr11G05652	BPChr11G05815	Tandem duplication
BPChr11G13446	BPChr11G13426	Tandem duplication
BPChr11G13441	BPChr11G13427	Tandem duplication
BPChr11G13438	BPChr11G13422	Tandem duplication
BPChr11G13445	BPChr11G13435	Tandem duplication
BPChr11G19172	BPChr11G19169	Tandem duplication

BPChr11G18472	BPChr11G18445	Tandem duplication
BPChr11G18408	BPChr11G18446	Tandem duplication
BPChr11G18446	BPChr11G18409	Tandem duplication
BPChr11G18409	BPChr11G18450	Tandem duplication
BPChr11G18450	BPChr11G18416	Tandem duplication
BPChr13G24633	BPChr13G24603	Tandem duplication
BPChr13G24580	BPChr13G24613	Tandem duplication
BPChr13G24613	BPChr13G24594	Tandem duplication
BPChr13G24594	BPChr13G24608	Tandem duplication
BPChr14G12639	BPChr14G12643	Tandem duplication
BPChr14G27023	BPChr14G26992	Tandem duplication
BPChr14G27062	BPChr14G27089	Tandem duplication
BPChr14G27089	BPChr14G26985	Tandem duplication
BPChr01G24963	BPChr03G02379	Segmental duplication
BPChr03G10929	BPChr08G07676	Segmental duplication
BPChr06G30381	BPChr06G30987	Segmental duplication
BPChr06G30771	BPChr13G24630	Segmental duplication
BPChr06G09558	BPChr14G12707	Segmental duplication
BPChr07G31997	BPChr11G18472	Segmental duplication
BPChr08G28436	BPChr08G28562	Segmental duplication
BPChr08G27453	BPChr11G06845	Segmental duplication
BPChr08G01527	BPChr11G26862	Segmental duplication
BPChr12G28184	BPChr14G26657	Segmental duplication
BPChr01G16946	BPChr09G04106	Segmental duplication
BPChr06G30788	BPChr06G30899	Segmental duplication
BPChr06G30608	BPChr06G31246	Segmental duplication
BPChr06G30671	BPChr06G31000	Segmental duplication
BPChr06G09493	BPChr08G15439	Segmental duplication
