# Supplementary material for: Characterization of the F-Box Gene Family and Its Expression under Osmotic Stress in Birch
Source: Plants (Basel). 2023 Nov 29;12(23):4018. doi: 10.3390/plants12234018 (PMC10707895; doi:10.3390/plants12234018)
Supplement: Supplementary file 1 [file plants-12-04018-s001.zip › Table S4.pdf]

Table S4 Syntenic gene pairs

| Gene ID | Gene ID |
|---|---|
| BPChr05G04622 | AT4G35733.1 |
| BPChr05G31582 | AT5G56180.1 |
| BPChr06G29343 | AT3G61590.1 |
| BPChr07G32029 | AT4G22390.1 |
| Potri.001G057100.1 | BPChr08G03093 |
| Potri.001G452000.1 | BPChr09G20612 |
| Potri.002G117900.1 | BPChr01G22922 |
| Potri.002G118700.1 | BPChr01G22906 |
| Potri.002G007700.1 | BPChr01G16908 |
| Potri.002G094600.1 | BPChr03G09664 |
| Potri.002G166500.1 | BPChr06G29343 |
| Potri.002G068200.1 | BPChr12G08200 |
| Potri.003G171300.1 | BPChr08G03093 |
| Potri.004G033900.1 | BPChr03G28832 |
| Potri.005G253500.1 | BPChr01G16908 |
| Potri.005G192300.1 | BPChr12G08200 |
| Potri.006G254100.1 | BPChr01G24963 |
| Potri.006G012900.1 | BPChr07G32029 |
| Potri.006G196900.1 | BPChr14G12944 |
| Potri.008G050800.1 | BPChr06G09558 |
| Potri.011G042400.1 | BPChr03G28832 |
| Potri.011G149900.1 | BPChr09G20612 |
| Potri.014G016200.1 | BPChr01G22906 |
| Potri.014G093200.1 | BPChr06G29343 |
| Potri.016G012600.1 | BPChr07G32017 |
| Potri.017G102300.2 | BPChr07G10044 |
| Potri.018G027700.1 | BPChr01G24963 |
