# Supplementary material for: Characterization of the F-Box Gene Family and Its Expression under Osmotic Stress in Birch
Source: Plants (Basel). 2023 Nov 29;12(23):4018. doi: 10.3390/plants12234018 (PMC10707895; doi:10.3390/plants12234018)
Supplement: Supplementary file 1 [file plants-12-04018-s001.zip › Table S5.pdf]

Table S5 Primer sequences for qRT-PCR

Name	5' primers	3' primers
BPChr13G00943	CACCGTAGAAAGAAATCTGG	CCTTCACAGCTTGCCAATTG
BPChr08G27453	CACTTTATCAAAATGCACCT	ACAACCCGTTACAAGAACC
BPChr08G28436	ATGGCCATGGCAAAGGAGCAA	GAACATCGGAGACCACGAAAG
BPChr14G12707	CAATACTGGCATGAGCCTCATC	CTGCAATCTGCTTTTGGTGGTC
Bp18S	ATCTTGGGTTGGGCAGATCG	CATTACTC CGATCCCGAAGG
