# Supplementary figures and images for: Characterization of the F-Box Gene Family and Its Expression under Osmotic Stress in Birch
Source: Plants (Basel). 2023 Nov 29;12(23):4018. doi: 10.3390/plants12234018 (PMC10707895; doi:10.3390/plants12234018)

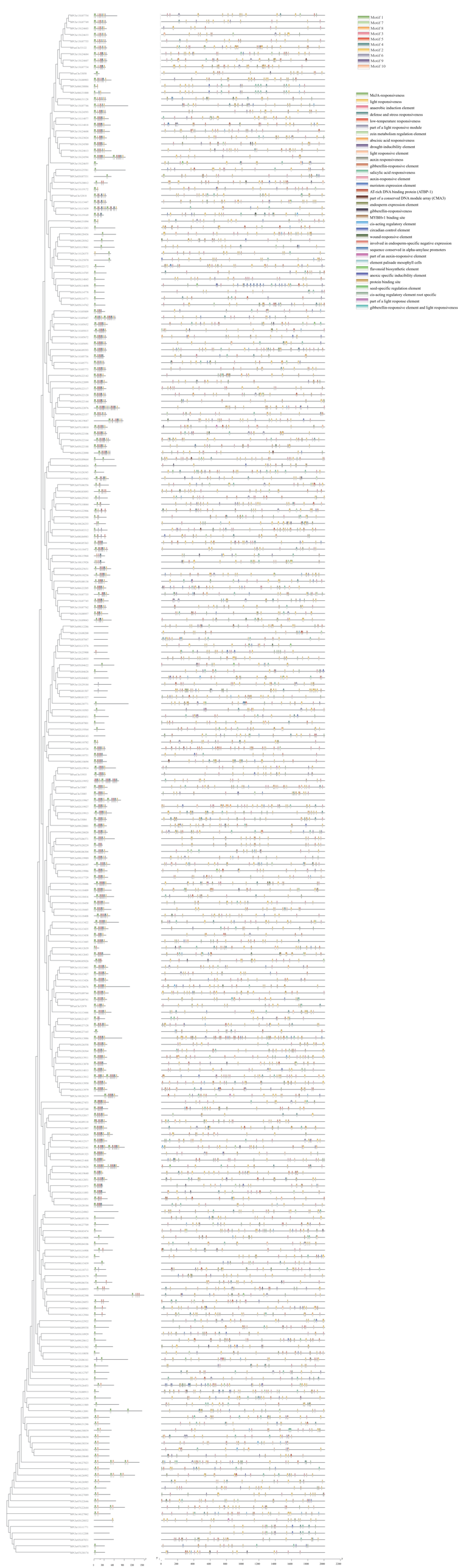

Supplement: Supplementary file 1 [file plants-12-04018-s001.zip › Figure S1.pdf]
